# Radiomics beyond oncology: new frontiers in thoracic imaging

**DOI:** 10.1590/0100-3984.2025.58.e4-en

**Published:** 2025-09-09

**Authors:** Marcel Koenigkam Santos

**Affiliations:** 1 Faculdade de Medicina de Bauru da Universidade de São Paulo (FMBRU-USP), Bauru, SP, Brasil.; 2 Faculdade de Medicina de Ribeirão Preto da Universidade de São Paulo (FMRP-USP), Ribeirão Preto, SP, Brasil. E-mail: marcelk46@fmrp.usp.br

In recent decades, we have witnessed a silent yet transformative revolution in the way we analyze medical imaging. The traditional subjective, qualitative evaluation of radiological images has been increasingly refined, leading toward an objective, quantitative analysis. In this context, radiomics, with its massive capacity to extract complex quantitative information from routine clinical exams, has been consolidating itself as one of the main tools in personalized, or precision, medicine. Although its applications were initially concentrated primarily in oncology, new directions are now appearing in several areas, such as diffuse bronchopulmonary diseases, including interstitial lung diseases.

Fitting within the context described above, there is the review article entitled “Radiomics in PET/CT and HRCT for systemic sclerosis-associated interstitial lung disease: breakthroughs and future directions” and recently published in **Radiologia Brasileira**^(^1^)^. The authors provided a comprehensive, up-to-date review of the applications of radiomics in two fundamental methods for the evaluation of interstitial lung disease associated with systemic sclerosis: high-resolution computed tomography and ^18^F-fluorodeoxyglucose positron-emission tomography/computed tomography.

The review highlights how radiomics, by integrating advanced artificial intelligence techniques, can refine the characterization of disease patterns on tomography, predict disease progression, identify metabolic biomarkers, and support decisions regarding personalized treatment plans. It should be borne in mind that these advances not only overcome the limitations inherent in making a diagnosis based on conventional visual analysis but also lead us toward the incorporation of objective, reproducible tools into the assessment of severity, prognosis, and clinical follow-up of patients.

In addition to the interesting, well-structured content on the specific topic of interstitial lung disease in systemic sclerosis, this review article also helps highlight another important aspect of recent advances: the recognition of the potential of radiomics outside the oncological context, in different imaging methods. Among those is the application of radiomics in the differentiation and stratification of idiopathic pulmonary fibrosis; in the quantitative assessment of emphysema in patients with chronic obstructive pulmonary disease; in the prediction of severity in viral pneumonias such as COVID-2019; and even in the characterization of vascular alterations in cases of pulmonary hypertension. Such studies demonstrate the potential of radiomics to provide objective biomarkers, which are especially useful in diseases with a chronic, heterogeneous course, in which the visual analysis can be limited or subjective.

It is also noteworthy that the advancement of radiomics depends on overcoming significant challenges. Such challenges include the standardization of image acquisition protocols; the homogenization of segmentation, extraction, and feature analysis methods; and the multicenter, prospective validation of predictive models. Overcoming these obstacles requires a collaborative effort between health care professionals and computer scientists in various countries, using different technologies to study diverse populations. In other words, we Brazilians have an important role to play in this, considering not only our regional leadership as a hub for scientific research in health but also our population diversity.

For readers who wish to delve deeper into the topic, in addition to this article published in **Radiologia Brasileira** and its references^(^1^)^, there are articles that discuss the use of radiomics outside the oncological context^(^2^,^3^)^, as well as other studies conducted in Brazil that deal with radiomics and other new tools for analyzing medical images^(^4^)^. They are all worth reading!

By publishing this review^(^1^)^, **Radiologia Brasileira** takes on an important role in fostering the dissemination of cutting-edge technical and scientific knowledge. May this article inspire new studies, collaborations, and clinical applications of radiomics in Brazil and in Latin America at large, contributing to more precise, predictive, and personalized medical practice.

## References

[1] Bastos AL, Mamede M (2025). Radiomics in PET/CT and HRCT for systemic sclerosis-associated
interstitial lung disease: breakthroughs and future
directions. Radiol Bras..

[2] Larue RTHM, Defraene G, De Ruysscher D (2017). Quantitative radiomics studies for tissue characterization: a
review of technology and methodological procedures. Br J Radiol..

[3] Fang Y, Zhang Q, Yan J (2025). Application of radiomics in acute and severe non-neoplastic
diseases: a literature review. J Crit Care..

[4] Koenigkam-Santos M, Ferreira JR, Wada DT (2019). Artificial intelligence, machine learning, computer-aided
diagnosis, and radiomics: advances in imaging towards to precision
medicine. Radiol Bras..

